# Dose-response and time-course effects of ecnoglutide in type 2 diabetes: a systematic review and meta-analysis of efficacy

**DOI:** 10.3389/fendo.2026.1934027

**Published:** 2026-09-11

**Authors:** Yixuan Feng, Changxin Sun, Xiaoya Li, Lanqing Hu, Kaidong Zhou, Lingling Li, Yongfang Yuan, Lili Yu, Min Wu, Longtao Liu

**Affiliations:** 1 Department of Cardiology, Guang’anmen Hospital, China Academy of Chinese Medical Sciences, Beijing, China; 2 Beijing University of Chinese Medicine, Beijing, China; 3 State Key Laboratory of Traditional Chinese Medicine Syndrome/National Clinical Research Center for Chinese Medicine Cardiology, Xiyuan Hospital, China Academy of Chinese Medical Science, Beijing, China

**Keywords:** ecnoglutide, GLP-1 receptor agonist, glycemic control, HbA1c, meta-analysis, safety, type 2 diabetes mellitus

## Abstract

**Background:**

Ecnoglutide, a novel long-acting GLP-1 receptor agonist, is considered a promising therapeutic option for the treatment of diabetes. This study aims to evaluate the efficacy and safety of ecnoglutide in adults with type 2 diabetes and to further investigate its dose-time relationship.

**Methods:**

This study followed the PRISMA guidelines and systematically searched PubMed, Web of Science, Embase, and Cochrane Library, with the search updated through February 2026. Randomized controlled trials comparing ecnoglutide with placebo or the active control, dulaglutide, were included. The primary outcome was the change in HbA1c (%). Secondary outcomes included FPG, PPG, 7-point SMBG, body weight, adverse events, and serious adverse events. Risk of bias was assessed using the Cochrane RoB-2 tool. Meta-analyses employed a random-effects model, and mean differences and risk ratios were calculated with 95% confidence intervals.

**Results:**

Three randomized controlled trials involving 977 participants were included. Compared with placebo, ecnoglutide significantly reduced HbA1c (MD = −1.40, 95% CI −1.65, −1.14, p < 0.00001), FPG (MD = −1.98, 95% CI −2.34, −1.63, p < 0.00001), PPG (MD = −4.88, 95% CI −6.32, −3.44, p < 0.00001), and body weight (MD = −2.16, 95% CI −2.62, −1.71, p < 0.00001). Additionally, compared with the placebo group, the incidence of adverse events was higher in the ecnoglutide group (OR = 2.03, 95% CI 1.24–3.32, p = 0.005), while there was no significant difference in the incidence of serious adverse events. Dose-response analysis showed that ecnoglutide exhibited a clear dose-dependent relationship for HbA1c, FPG, and body weight at doses ranging from 0.4 to 1.2 mg, while PPG was less affected by dose. Time-response analysis indicated that the hypoglycemic efficacy of ecnoglutide peaked around week 35 and subsequently plateaued, whereas body weight exhibited a sustained linear downward trend.

**Conclusion:**

Ecnoglutide significantly improved glycemic control and reduced body weight, although it was associated with a higher incidence of adverse events without increasing serious adverse events. Despite these promising findings, the evidence remains limited and requires confirmation in larger, multicenter randomized controlled trials.

**Systematic review registration:**

https://www.crd.york.ac.uk/PROSPERO/view/CRD420261327235, identifier CRD420261327235.

## 1 Introduction

The global prevalence of diabetes continues to rise; approximately 537 million adults worldwide have diabetes, and 6.7 million people die each year from diabetes-related chronic kidney disease, cardiovascular disease, and microvascular complications (1). The vast majority of these cases are type 2 diabetes; the number of people with the disease is projected to rise to 783 million by 2045 (2), while the global prevalence of undiagnosed diabetes is only 45%. In addition, approximately 352 million people have impaired fasting glucose or impaired glucose tolerance, and 5%–10% of this population progresses to type 2 diabetes each year (3, 4). Although new cases of type 2 diabetes increase rapidly after age 55, the incidence of early-onset type 2 diabetes among people under 40 continues to rise, posing new public health and social challenges (5).

Although there is a wide variety of antidiabetic drugs available, the rate of achieving target blood glucose levels among people with type 2 diabetes worldwide remains below 50 percent. Furthermore, poor adherence, treatment inertia, and discontinuation of therapy due to adverse reactions are common issues during long-term treatment (6, 7). GLP-1 receptor agonists have revolutionized the treatment of diabetes by simultaneously improving both glycemic control and metabolic abnormalities in patients with type 2 diabetes. Through mechanisms such as glucose-dependent insulin secretion, inhibition of glucagon secretion, delayed gastric emptying, and appetite regulation, these drugs can significantly reduce HbA1c levels and body weight while improving cardiovascular outcomes (8–10). Ecnoglutide, a novel cAMP-biased GLP-1 receptor agonist, is designed to improve tolerability while maintaining potent hypoglycemic and weight-loss effects. It has demonstrated good efficacy and safety in clinical trials, features a longer half-life, is less susceptible to degradation by dipeptidyl peptidase, and exhibits superior receptor selectivity, offering significant advantages over marketed drugs in this class, such as semaglutide and dulaglutide (11–13). The drug is currently still in the clinical trial phase, and the ongoing Phase II and Phase III clinical trials continue to provide further evidence, fully confirming its clinical potential.

To date, studies on the use of ecnoglutide for the treatment of type 2 diabetes have not systematically elucidated the relationship between different dosages and timing of administration and glycemic control. Based on this, the purpose of this study is to comprehensively evaluate the clinical efficacy of ecnoglutide in adult patients with type 2 diabetes by comparing it with a positive control drug and a placebo. The study uses glycated hemoglobin as the primary outcome measure and fasting blood glucose, postprandial blood glucose, and the incidence of adverse events as secondary outcome measures to investigate the clinical value of ecnoglutide in the treatment of type 2 diabetes.

## 2 Methods

### 2.1 Protocol and registration

This systematic review and meta-analysis were conducted in strict accordance with the PRISMA (Preferred Reporting Items for Systematic Reviews and Meta-Analyses) guidelines (14). The study protocol has been pre-registered in the PROSPERO database under registration number CRD420261327235.

### 2.2 Search strategy

This study conducted a systematic search of the PubMed, Embase, Web of Science, and The Cochrane Library databases, covering the period from the inception of each database to April 1, 2026, with no language restrictions during the search phase. However, only English-language literature was included in the full-text screening and analysis stages. The search covered terms related to “ecnoglutide” and “diabetes” (e.g., ecnoglutide, diabetes, HbA1c). The complete search strategy (including search terms and methods) is provided in **Supplementary Table 1**. At the same time, we manually screened the reference lists of the included studies and relevant references to identify additional potentially relevant studies. Two researchers independently screened the titles, abstracts, and full texts of the studies; any disagreements were resolved through discussion or consultation with a third researcher.

### 2.3 Study selection

Studies must meet the following criteria for inclusion: (a) participants must be adults with type 2 diabetes; (b) the intervention must be ecnoglutide; (c) a control group must be included (e.g., open-label control, placebo control, active drug control); (d) The intervention period must be at least 6 weeks; (e) The study must be a randomized controlled trial (RCT) published in a peer-reviewed English-language journal.

### 2.4 Data extraction

Data were independently extracted by two researchers using a standardized template. The extracted information included: basic study information (authors, year of publication, study design, clinical trial registration number, sample size); participant demographics (mean age, gender, duration of diabetes); intervention details (ecnoglutide dose, duration of intervention); control measures (type of control, dose, duration); outcome measures (primary outcomes: including indicators related to blood glucose and insulin regulation, such as glycated hemoglobin (HbA1c), fasting plasma glucose (FPG), 2-hour postprandial glucose, and 7-point self-monitored blood glucose (SMBG). Secondary outcomes included anthropometric measures: body weight. Safety measures include all adverse events, serious adverse events, and adverse events leading to discontinuation). Disagreements were resolved through discussion with a third researcher and/or the senior corresponding author. For any uncertain data, the original study authors were contacted to obtain subgroup data to enable multiple comparisons of different treatment strategies.

### 2.5 Risk of bias assessment

Two researchers independently assessed the risk of bias in the included studies using the Cochrane Risk of Bias 2.0 tool (RoB 2) (15). Any disagreements between the two assessors were resolved through consultation with a third researcher.

### 2.6 Statistical analysis

Statistical analysis was performed using Review Manager (RevMan 5.4) and Stata 19.5, with a significance level of α = 0.05. The statistical methods applied in this systematic review and meta-analysis were selected according to established methodological recommendations for evidence synthesis and meta-analytic studies (16). Continuous outcome measures were reported as mean differences (MD) with 95% confidence intervals (CI), while dichotomous outcome measures were reported as odds ratios (OR) with 95% CI. Data were pooled using a random-effects model to account for expected heterogeneity among studies; a fixed-effects model was also included for comparison. The I² statistic was used to assess heterogeneity among studies (17); an I² value greater than 50% indicates significant heterogeneity. A leave-one-out sensitivity analysis was performed to evaluate the robustness of the pooled estimates. Potential sources of heterogeneity were explored through subgroup analyses according to comparator (placebo versus dulaglutide), where sufficient data were available. Because only three randomized controlled trials were included, additional subgroup analyses based on study characteristics or patient demographics were not feasible.

To evaluate the dose-response relationship of ecnoglutide, inverse-variance weighted regression analysis was performed using treatment dose (0.4, 0.6, 0.8, and 1.2 mg/week) as a continuous independent variable and the corresponding treatment effect as the dependent variable. Inverse-variance weighting was applied to assign greater weight to effect estimates with higher precision. This approach was based on established methods for estimating dose-response relationships from summarized study-level data in meta-analyses (18).

Time-course effects of ecnoglutide were assessed using inverse-variance weighted polynomial regression, with treatment duration entered as a continuous variable. A quadratic term was incorporated to allow for potential non-linear changes in treatment effects over time, and predicted effect curves with corresponding 95% confidence intervals were generated from the fitted regression model. Because repeated observations from the same study may be correlated, the time-course analysis was considered exploratory and aimed to characterize overall temporal patterns rather than to provide formal inferential estimates.

## 3 Results

### 3.1 Study selection

**Figure 1:**
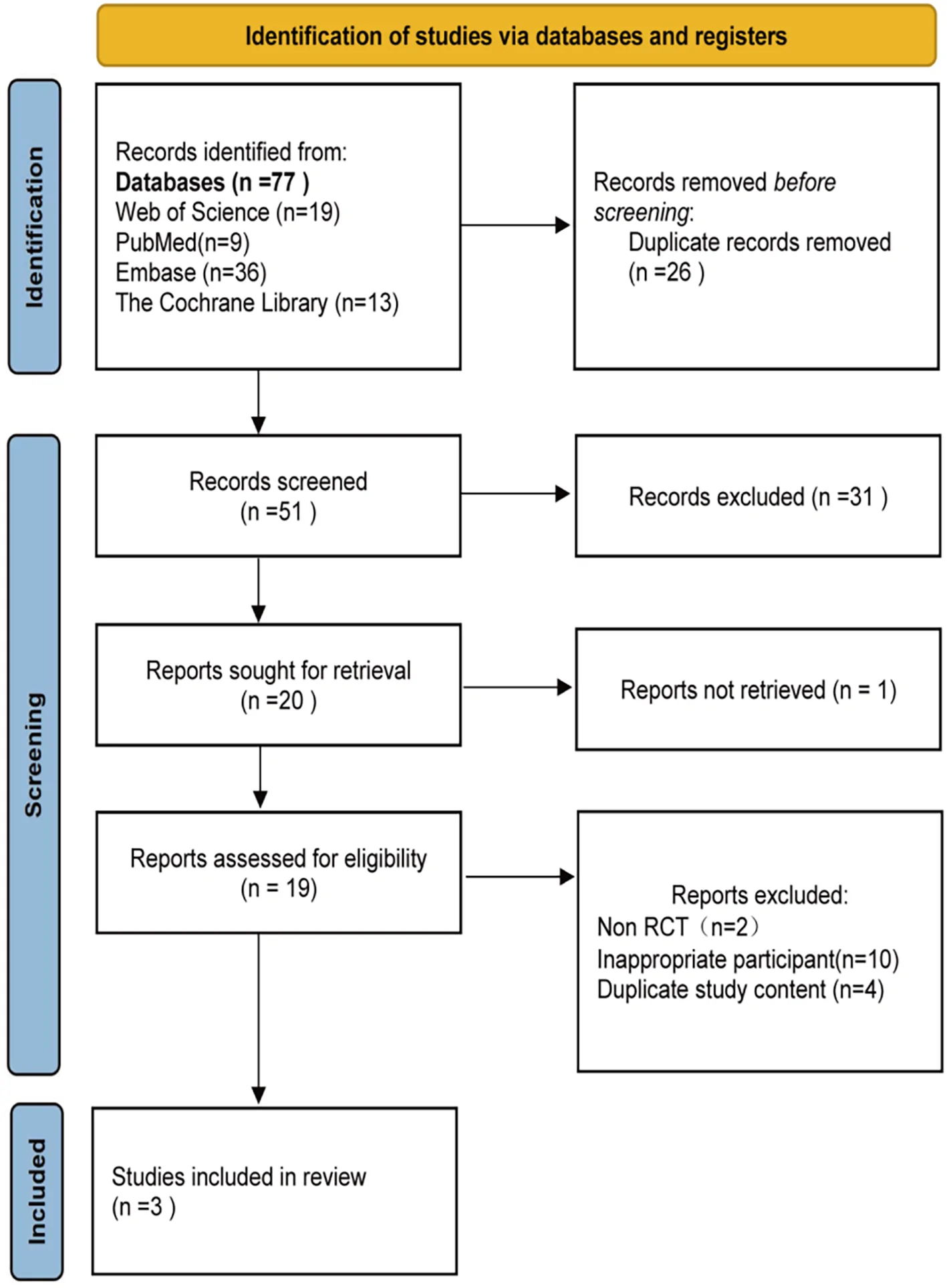
Study selection flow diagram.

A total of 77 articles were identified through database searches. After excluding duplicates, 51 potentially relevant articles were selected for abstract screening. During the full-text screening phase, 19 articles were reviewed, of which 16 were excluded for failing to meet the inclusion criteria (**Supplementary Table 2**). Ultimately, three studies were included for systematic review and quantitative meta-analysis (**Figure 1**).

### 3.2 Study characteristics

This review included a total of 977 participants, comprising 663 in the intervention group and 314 in the control group. The proportion of women ranged from 22.2% to 47.2%; the mean age of participants was 49.1–54.2 years; the mean duration of type 2 diabetes was 36–76.2 months; the ecnoglutide dose ranged from 0.4 to 1.2 mg; and the intervention duration ranged from 20 to 52 weeks (**Table 1**).

**Table 1:** Characteristics of the included randomized controlled trials evaluating the efficacy and safety of ecnoglutide in patients with type 2 diabetes.

Author/Year	Sample size	Mean age/male %	Duration of diabetes (months)	HbA1c(%)	BMI (kg/m²)	Intervention group	Control group	Treatment cycle (week)
Yang H, 2025 (12)	N=621 Ecnoglutide 0.6 mg: 206 Ecnoglutide 1.2 mg: 208 Dulaglutide 1.5 mg: 207	53.9/56	63.6–76.8	8.40	26.9	Ecnoglutide 0.6 mg or 1.2 mg	Dulaglutide 1.5 mg	52
Zhu D, 2024 (11)	N=145 Ecnoglutide 0.4 mg: 37 Ecnoglutide 0.8 mg: 36 Ecnoglutide 1.2 mg: 36 Placebo: 36	49.7–51.8/52.8–77.8	40–58	8.44–8.67	25.8–26.6	Ecnoglutide 0.4 mg、0.8 mg or 1.2 mg	Placebo (Ecnoglutide diluent), once weekly, subcutaneous injection	20
Zhu D,2026 (13)	N=211 Ecnoglutide 0.6 mg: 69 Ecnoglutide 1.2 mg: 71 Placebo: 71	52/60	43	8.52%	26.93	Ecnoglutide 0.6 mg or 1.2 mg	Placebo (volume-matched), once weekly, subcutaneous injection	24

### 3.3 Risk of bias assessment

The results of the risk of bias assessment showed that two studies were rated as having a low risk of bias (11, 13), while one study raised some concerns regarding bias due to its open-label design (12). Detailed assessment results are presented in **Supplementary Table 3**.

### 3.4 Efficacy outcomes

**Figure 2:**
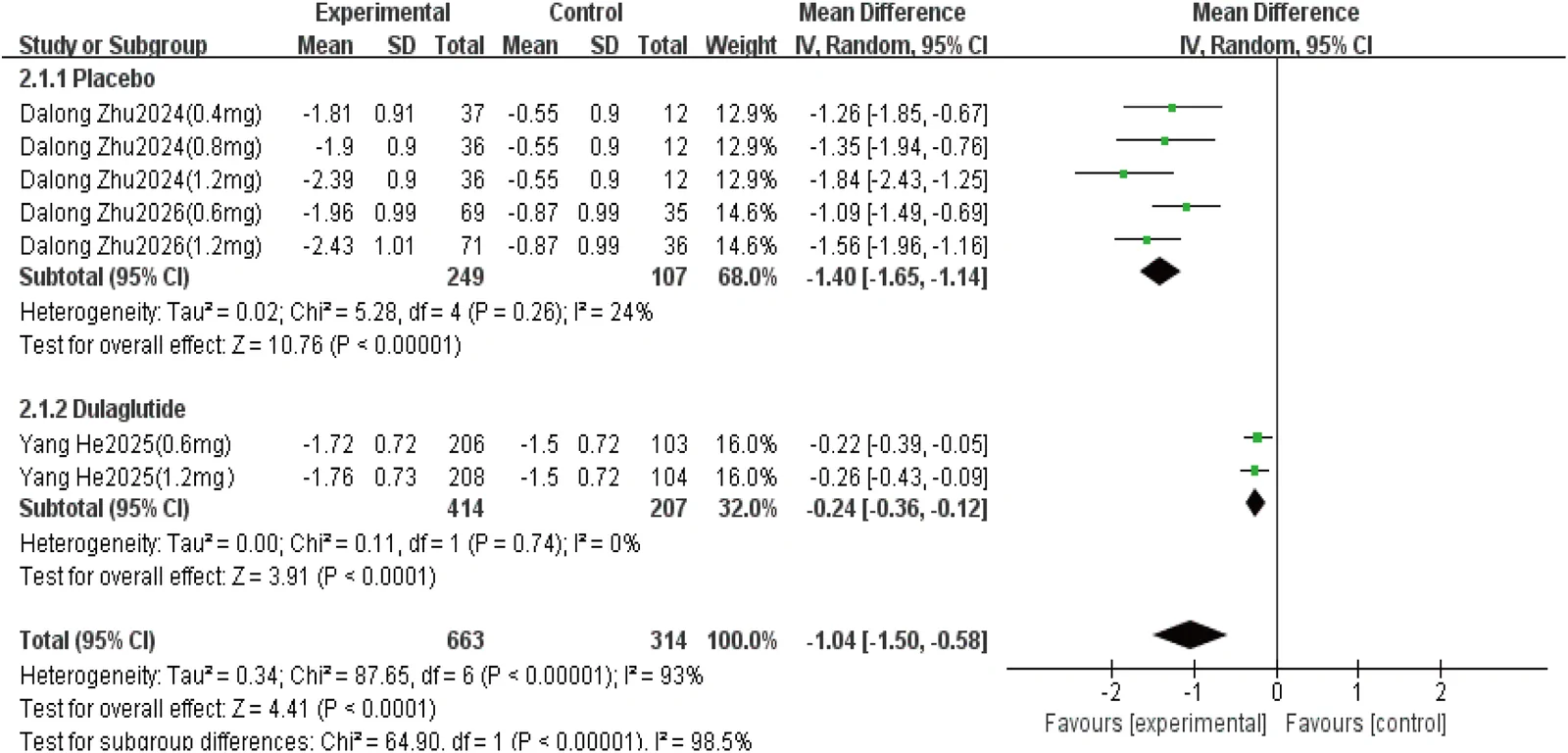
Forest plot of change in HbA1c (%).

The results of the pooled analysis of the included studies showed that ecnoglutide significantly improved various glycemic control parameters (**Figure 2**). HbA1c (%) levels decreased significantly from baseline: in the placebo-controlled group, MD = -1.40 (95% CI -1.65 to -1.14, p < 0.00001); the dulaglutide-controlled group showed an MD of −0.24 (95% CI −0.36 to −0.12, p < 0.0001).

**Figure 3:**
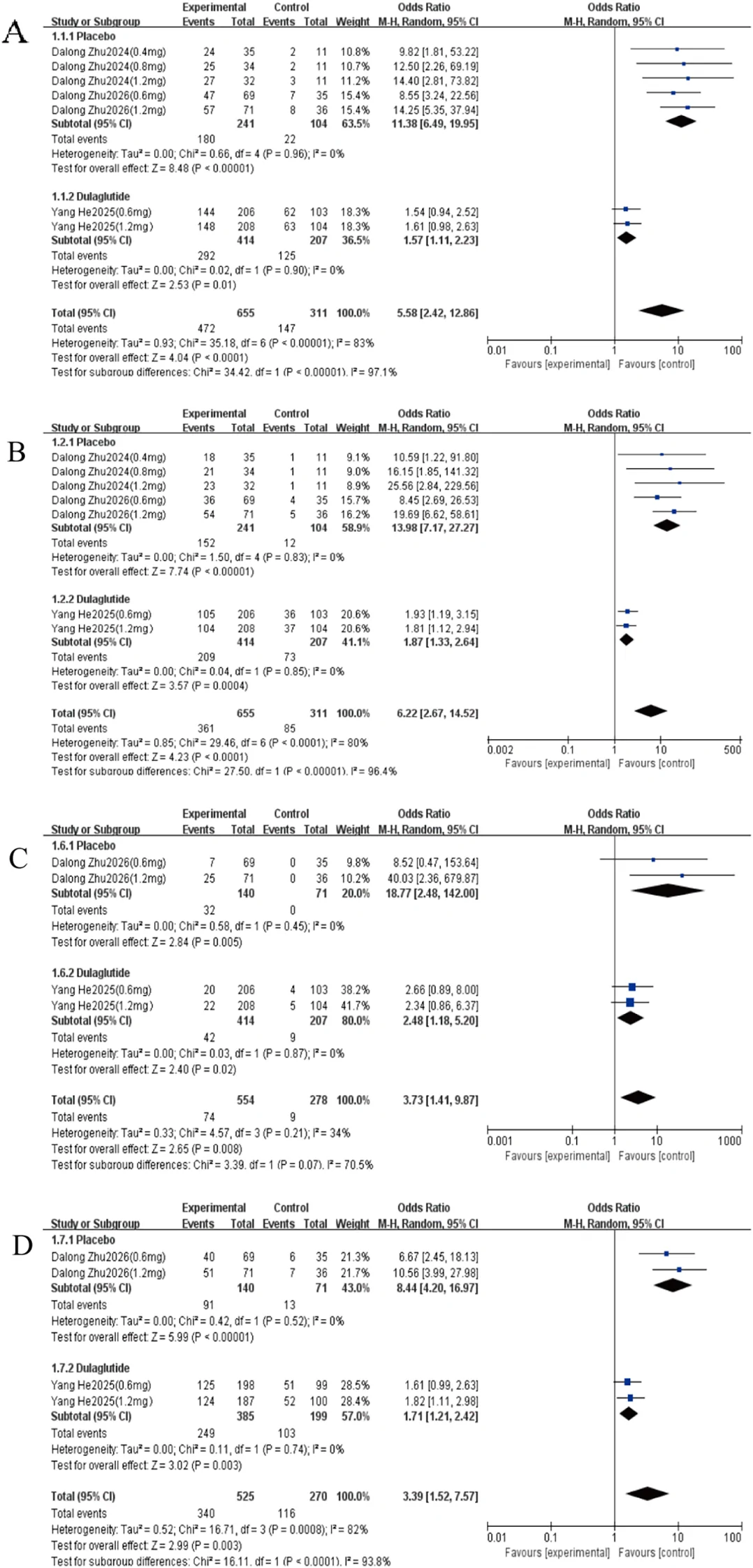
Forest plot of participants achieving HbA1c targets. **(A)** Achievement of HbA1c target <7.0%; **(B)** achievement of HbA1c target <6.5%; **(C)** achievement of HbA1c target <5.7%; **(D)** achievement of composite treatment goal of HbA1c <7.0% without hypoglycemia or weight loss.

Compared with the control group, participants in the ecnoglutide group had a higher rate of achieving HbA1c targets (**Figure 3**). The odds ratio (OR) for HbA1c < 7.0% in the placebo-controlled group was 11.38 (95% CI 6.49 to 19.95, p < 0.00001); the OR for the dulaglutide control group was 1.57 (95% CI 1.11 to 2.23, p = 0.01). For HbA1c < 6.5%, the OR in the placebo-controlled group was 13.98 (95% CI 7.17 to 27.27, p < 0.00001); in the dulaglutide-controlled group, the OR was 1.87 (95% CI 1.33 to 2.64, p = 0.0004). For HbA1c < 5.7%, the OR in the placebo-controlled group was 18.77 (95% CI 2.48 to 142.00, p = 0.005); in the dulaglutide-controlled group, the OR was 2.48 (95% CI 1.18 to 5.20, p = 0.02). For HbA1c < 7.0% with no hypoglycemia or weight loss, the OR in the placebo-controlled group was 8.44 (95% CI 4.20 to 16.97, p < 0.00001); in the dulaglutide-controlled group, the OR was 1.71 (95% CI 1.21 to 2.42, p = 0.003).

**Figure 4:**
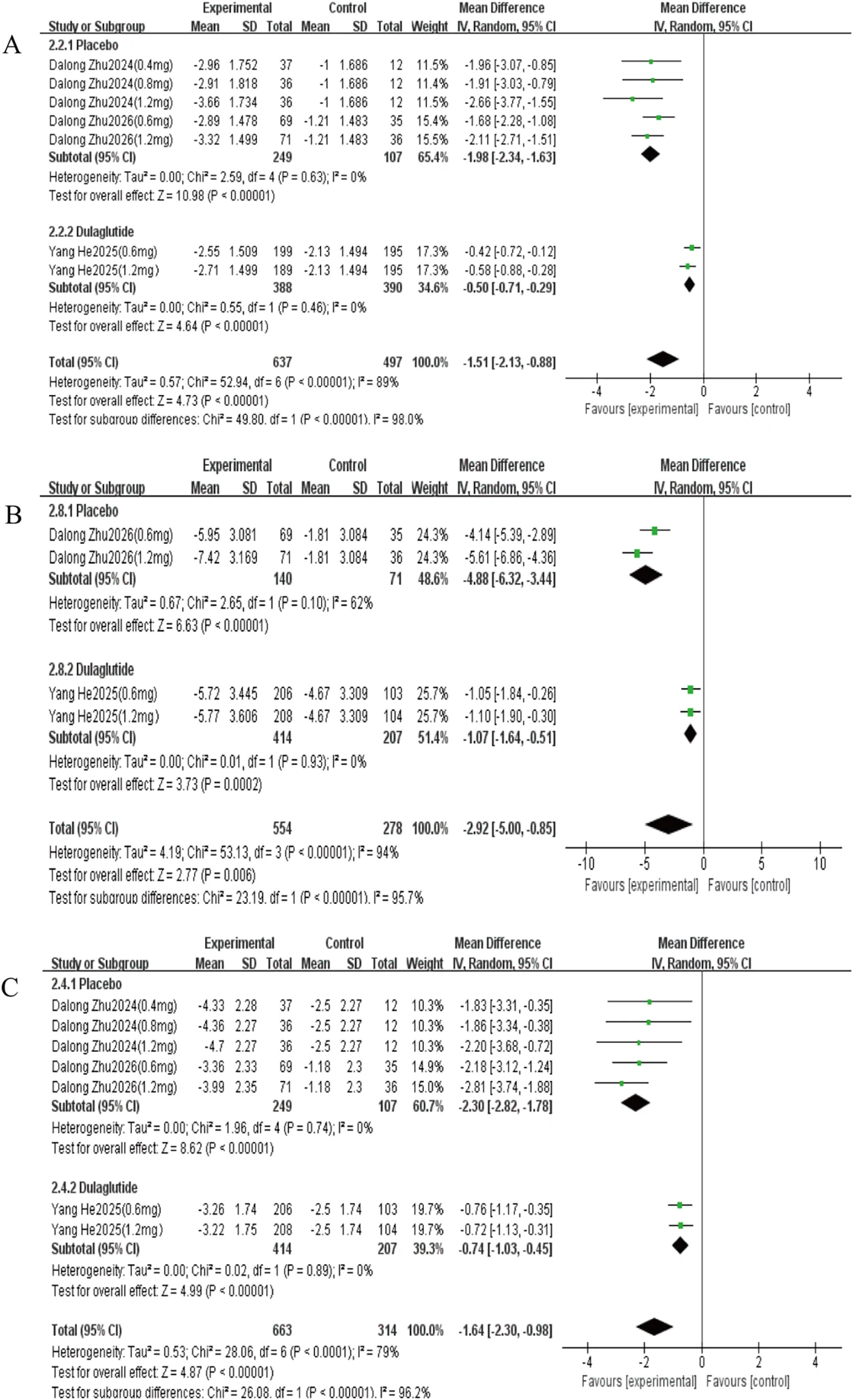
Forest plot of fasting plasma glucose and Postprandial Plasma Glucose (mmol/L). **(A)** Effect of ecnoglutide on fasting blood glucose (FPG); **(B)** effect of ecnoglutide on two-hour postprandial glucose (2-h PPG); **(C)** effect of ecnoglutide on seven-point self-monitored blood glucose (SMBG).

Fasting blood glucose levels were significantly reduced (**Figure 4**): MD = -1.98 (95% CI -2.34 to -1.63, p < 0.00001) in the placebo-controlled group; MD = -0.50 (95% CI -0.71 to -0.29, p < 0.00001) in the dulaglutide-controlled group. Two-hour postprandial glucose levels also decreased: MD = −4.88 (95% CI −6.32 to −3.44, p < 0.00001) in the placebo control group; MD = −1.07 (95% CI −1.64 to −0.51, p = 0.0002) in the dulaglutide control group. Seven-point SMBG levels also decreased significantly: MD = -2.30 (95% CI -2.82 to -1.78, p < 0.00001) in the placebo control group; MD = -0.74 (95% CI -1.03 to -0.45, p < 0.00001) in the dulaglutide control group.

### 3.5 Safety outcomes

**Figure 5:**
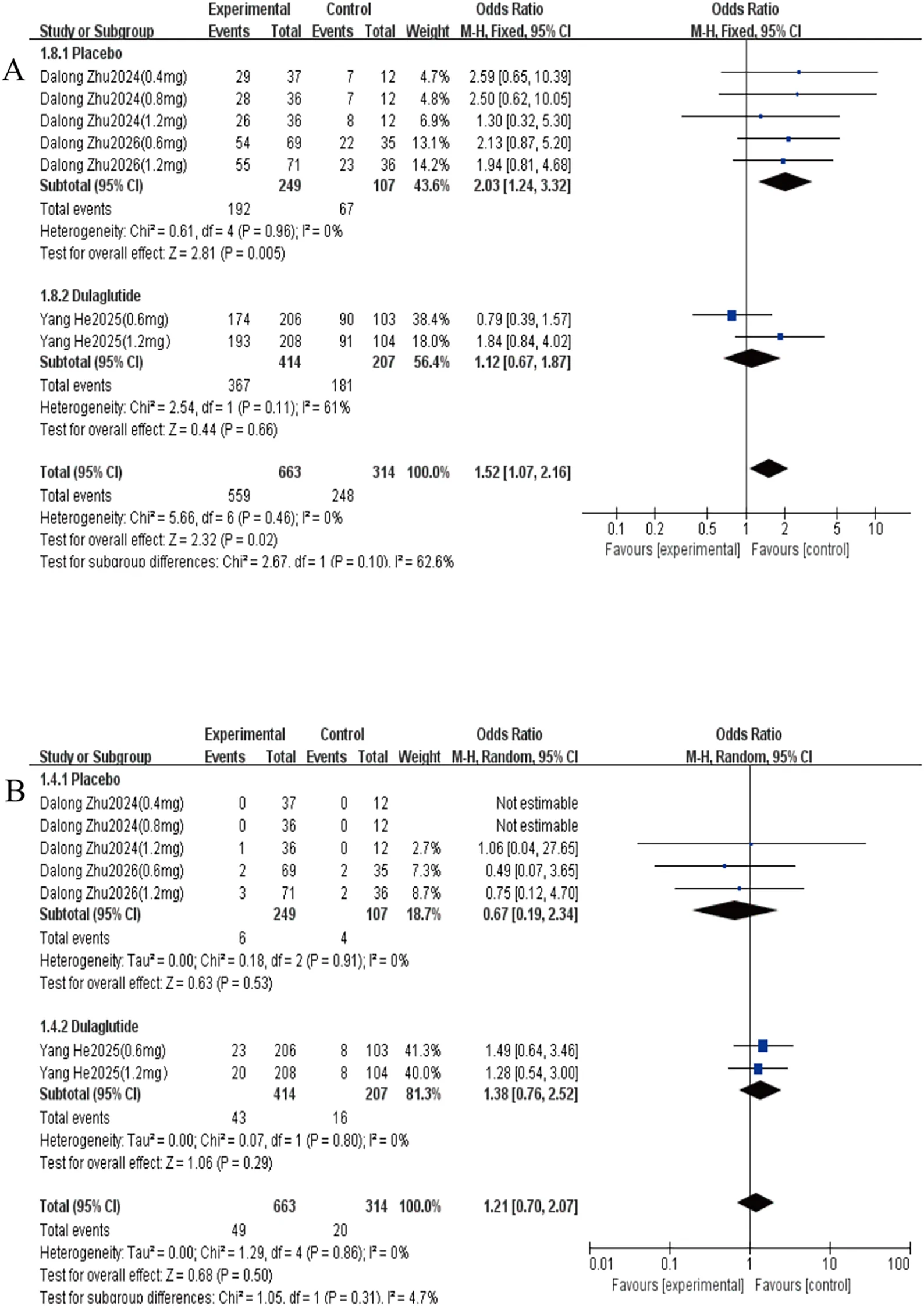
Forest plot of adverse events and serious adverse events of ecnoglutide. **(A)** Effect of ecnoglutide on the incidence of adverse events; and **(B)** effect of ecnoglutide on the incidence of serious adverse events.

The incidence of adverse events was higher in the ecnoglutide group compared with the placebo group (OR = 2.03, 95% CI 1.24 to 3.32, p = 0.005). For serious adverse events, the OR in the placebo-controlled group was 0.67 (95% CI 0.19 to 2.34, p = 0.53), and in the dulaglutide-controlled group, it was 1.38 (95% CI 0.76 to 2.52, p = 0.29); there was no statistically significant difference in incidence between the two groups (**Figure 5**).

### 3.6 Dose-response relationships of ecnoglutide

**Figure 6:**
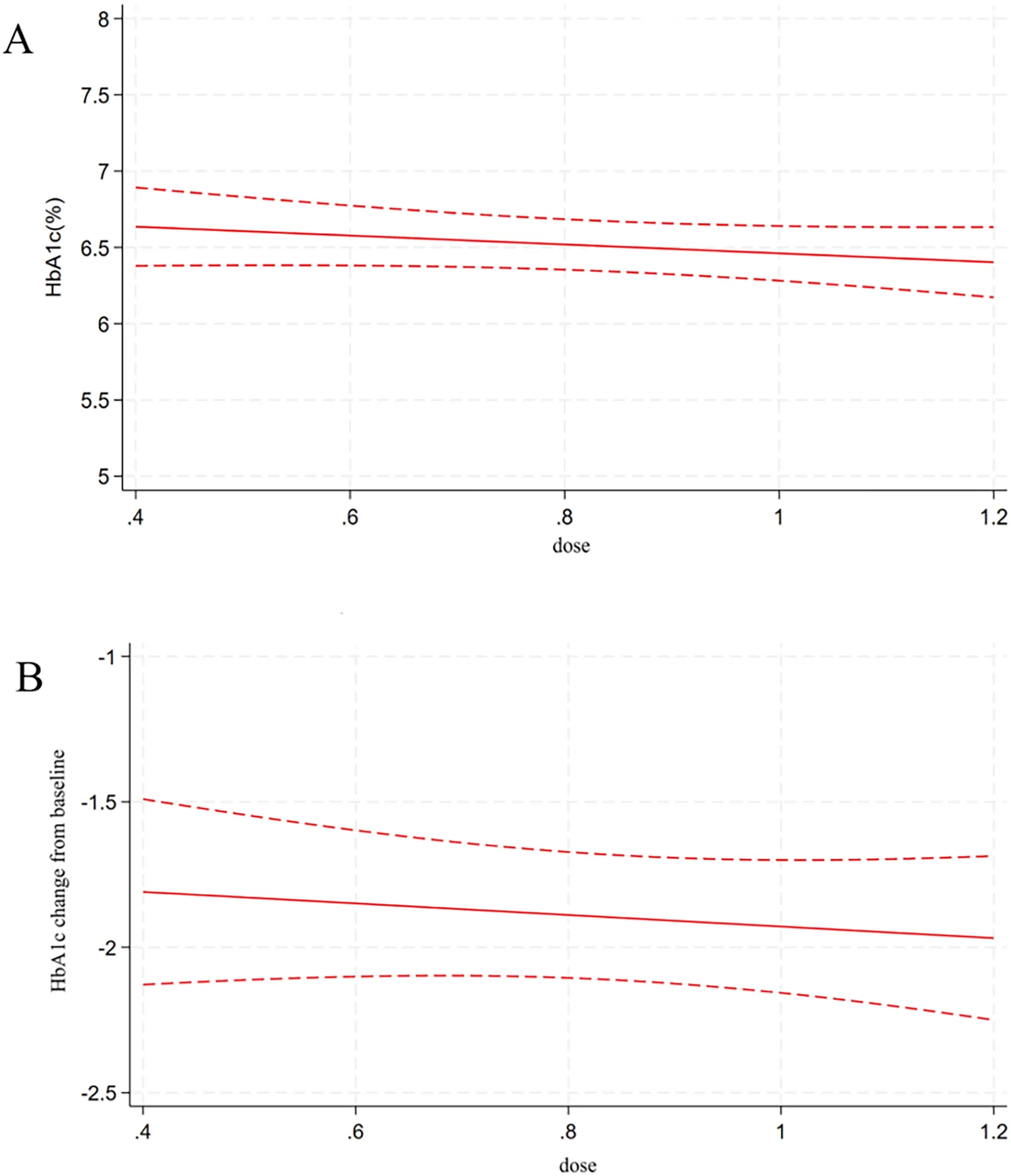
Dose-response relationships between ecnoglutide dose and glycemic control outcomes. **(A)** Dose-response curve showing the association between ecnoglutide dose (0.4–1.2 mg/week) and HbA1c levels; **(B)** dose-response curve showing the association between ecnoglutide dose (0.4–1.2 mg/week) and changes in HbA1c from baseline.

**Figure 7:**
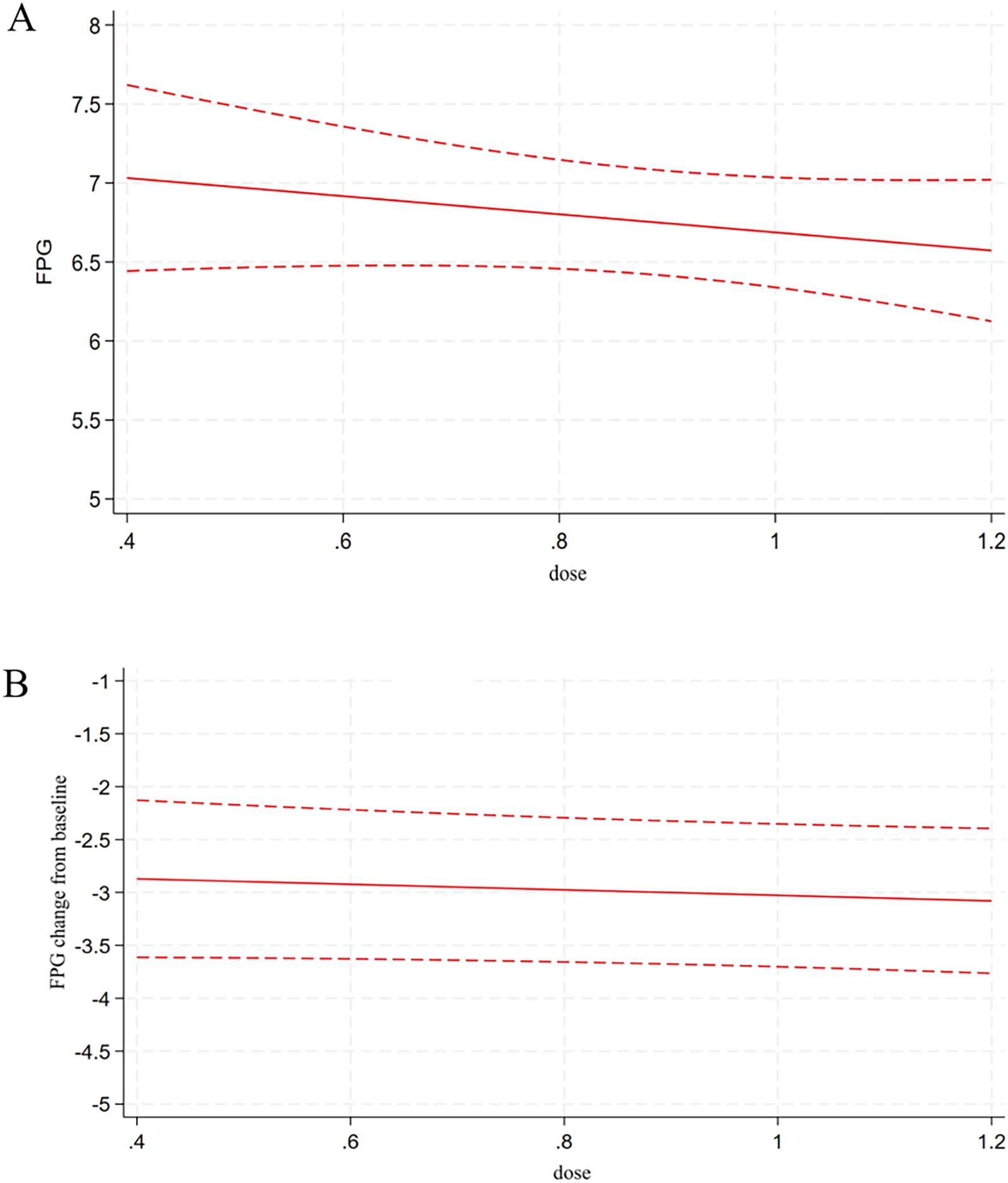
Dose-response relationships between ecnoglutide dose and fasting plasma glucose (FPG) outcomes. **(A)** Dose-response curve showing the association between ecnoglutide dose (0.4–1.2 mg/week) and FPG levels; **(B)** dose-response curve showing the association between ecnoglutide dose (0.4–1.2 mg/week) and changes in FPG from baseline.

**Figure 8:**
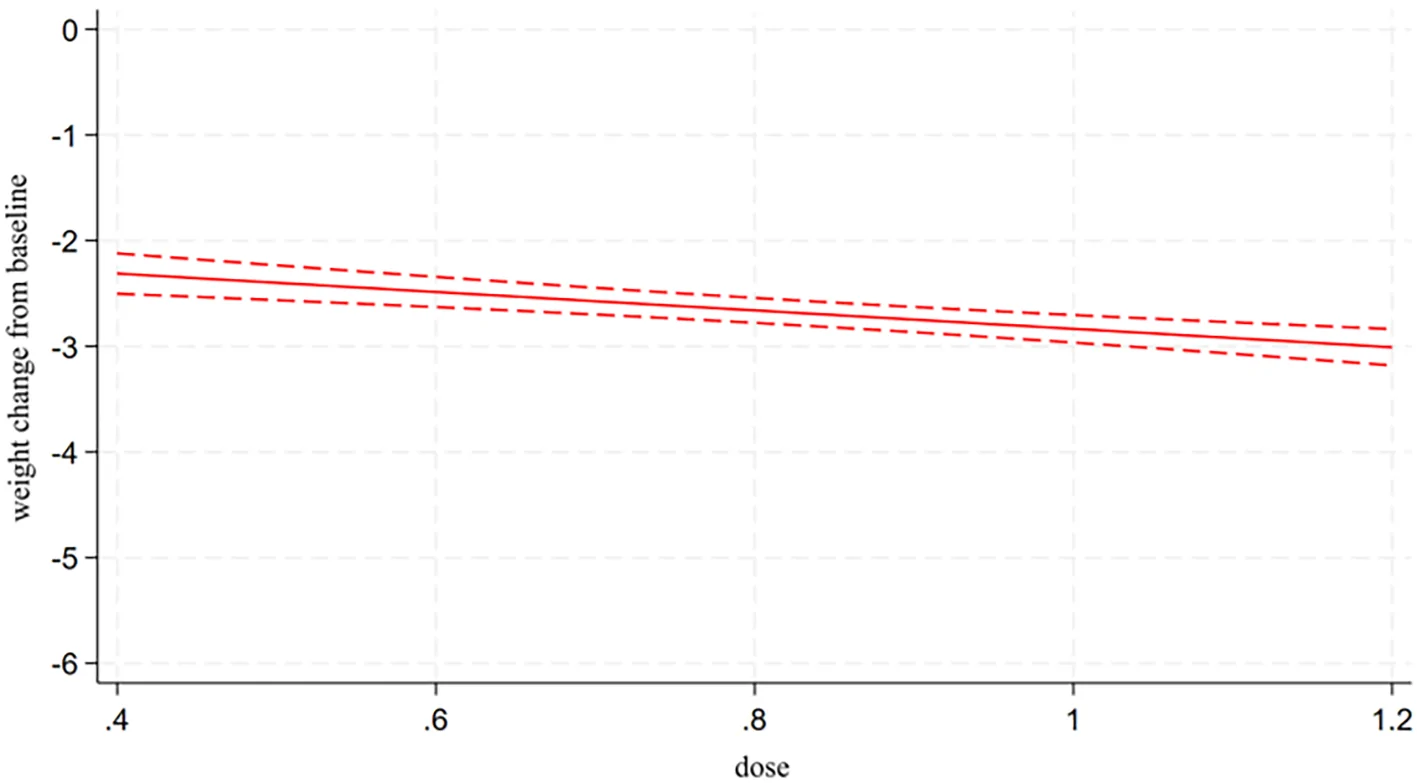
Dose-response relationship between ecnoglutide dose and weight change from baseline.

To investigate potential dose-response relationships, this study analyzed various outcome measures according to different doses of ecnoglutide (0.4 mg, 0.6 mg, 0.8 mg, 1.2 mg). Compared with the placebo-controlled group, ecnoglutide significantly reduced HbA1c levels, with the most pronounced reduction observed at the 1.2 mg dose (**Figure 6**). At the same time, indicators such as fasting plasma glucose (FPG) and percentage weight loss all exhibited a clear dose-response relationship, with the greatest reductions observed in the high-dose group (**Figures 7**, **8**).

**Figure 9:**
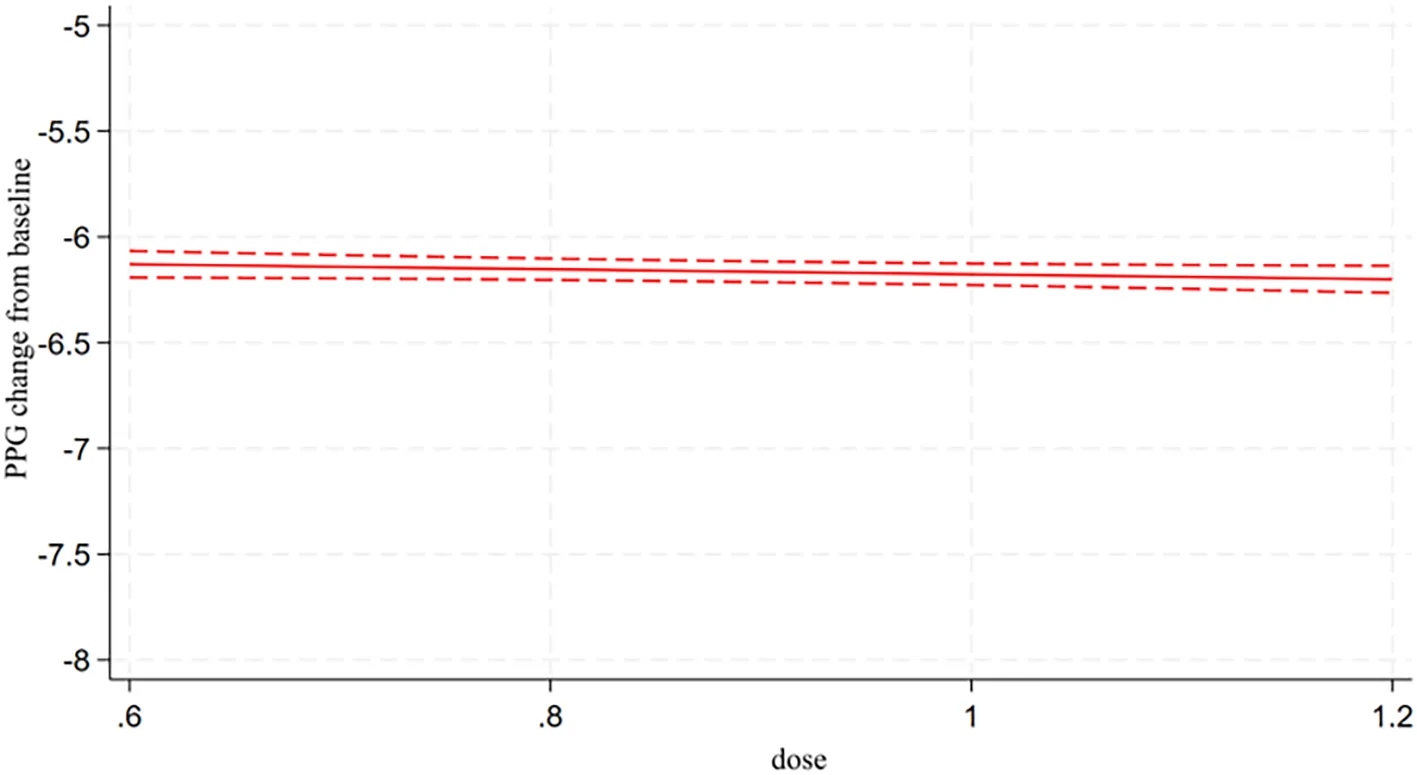
Dose-response relationship between ecnoglutide dose and PPG change from baseline.

In contrast, some parameters were less affected by dose or did not show a clear dose-response trend. There were no significant differences in 2-hour postprandial blood glucose levels among the different dose groups, and the differences between the low- and high-dose groups were not statistically significant. This suggests that the intensity of ecnoglutide’s effect on 2-hour postprandial blood glucose levels did not vary significantly with dose, and both the low and high doses achieved similar efficacy (**Figure 9**).

### 3.7 Time-response relationships of ecnoglutide

**Figure 10:**
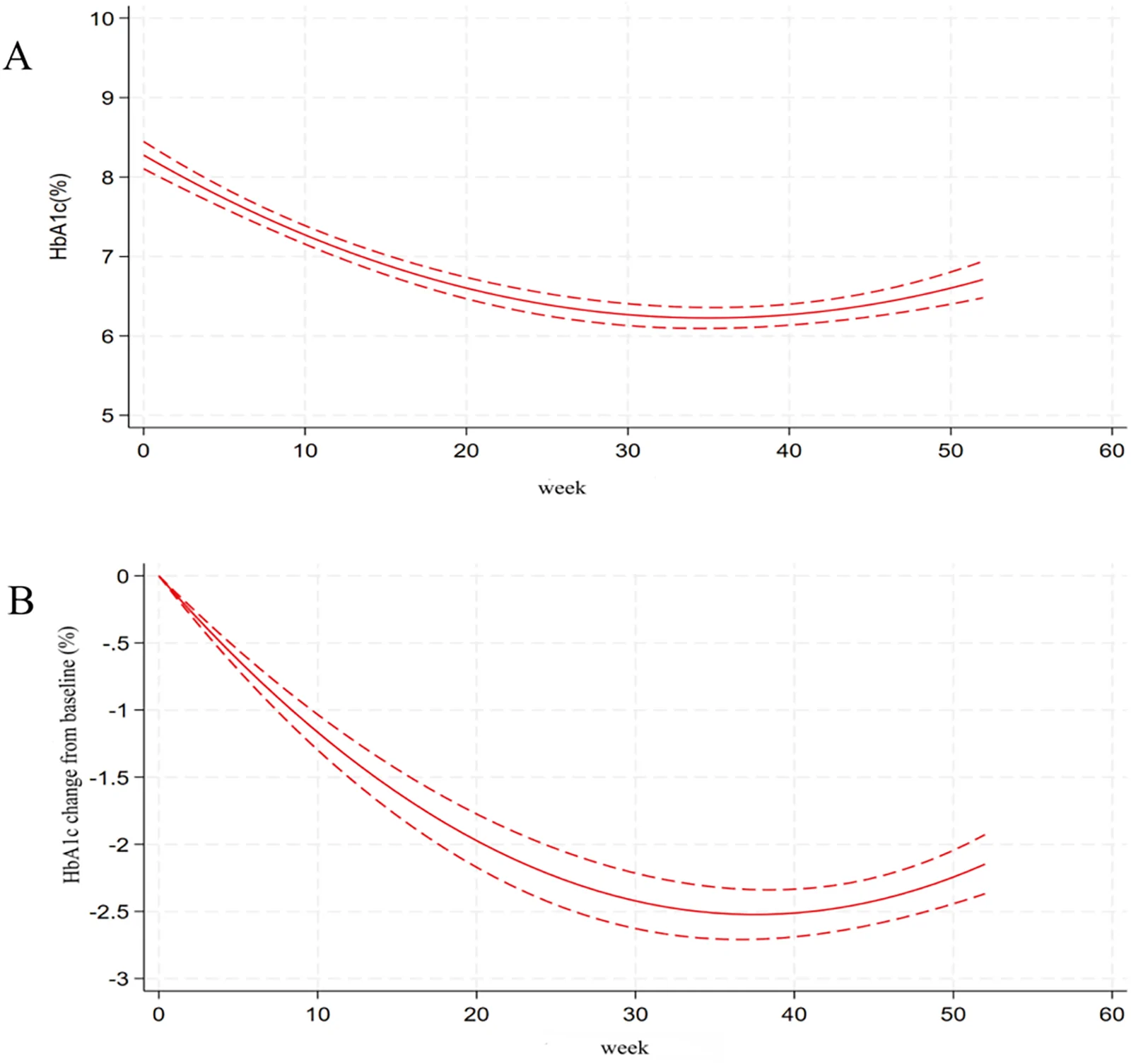
Time-course effects of ecnoglutide treatment on HbA1c outcomes during follow-up. **(A)** Time-course curve of HbA1c levels over follow-up weeks; **(B)** time-course curve of HbA1c change from baseline over follow-up weeks.

**Figure 11:**
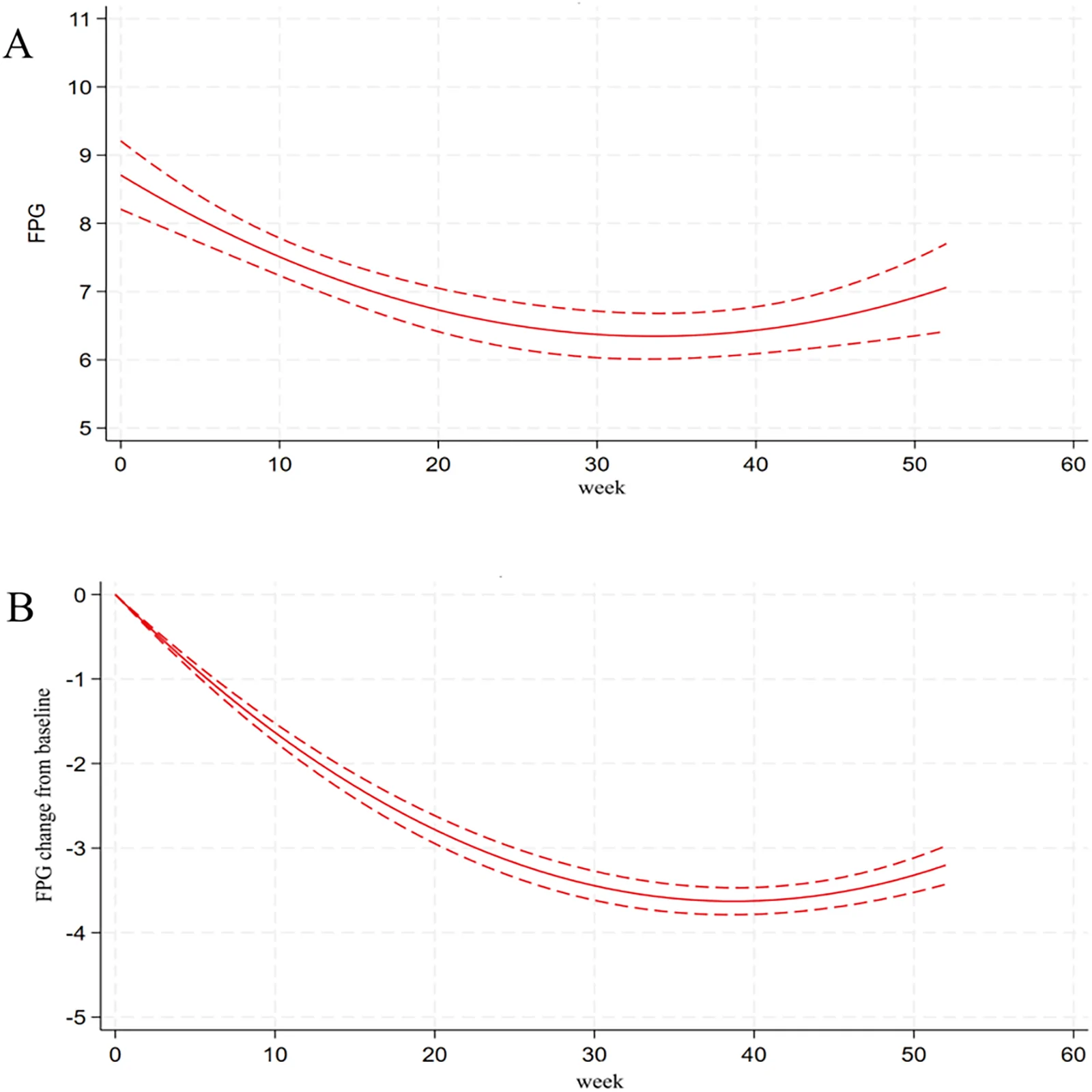
Time-course effects of ecnoglutide treatment on FPG outcomes during follow-up. **(A)** Time-course curve of FPG levels over follow-up weeks; **(B)** time-course curve of FPG change from baseline over follow-up weeks.

**Figure 12:**
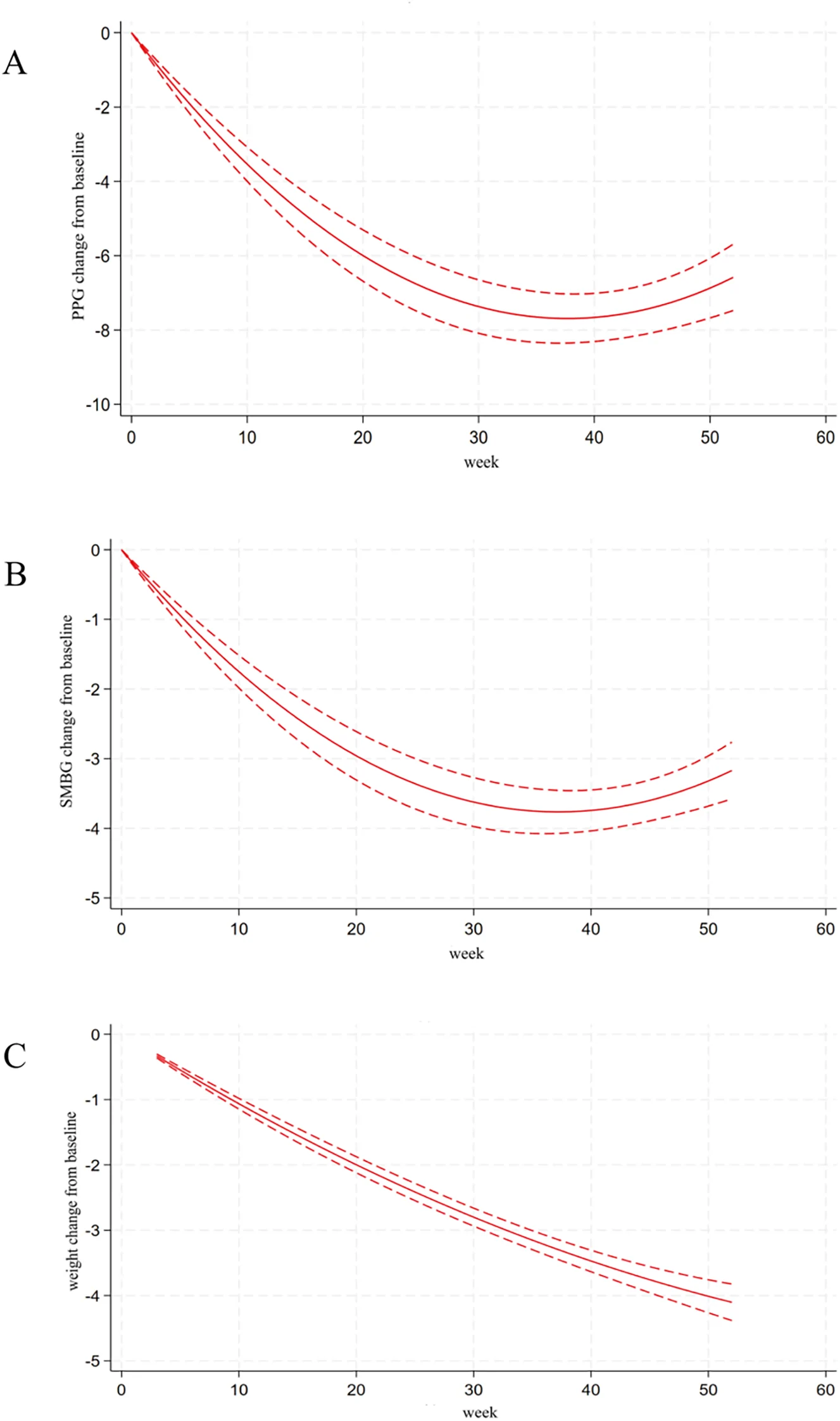
Time-course effects of ecnoglutide treatment on postprandial glucose, self-monitored blood glucose, and body weight outcomes during follow-up. **(A)** Time-course curve of two-hour postprandial glucose (2-h PPG) over follow-up weeks; **(B)** time-course curve of seven-point self-monitored blood glucose (SMBG) over follow-up weeks; and **(C)** time-course curve of body weight change from baseline over follow-up weeks.

To investigate the time-effect relationship, this study analyzed changes in indicators at different time points during the intervention. The results indicated that the therapeutic effects of ecnoglutide varied according to treatment duration. The findings revealed that the effects of ecnoglutide on HbA1c, FPG, PPG, and 7-point self-monitored blood glucose (SMBG) varied over time. Overall, these parameters demonstrated a nonlinear time-dependent pattern, characterized by an initial progressive reduction followed by stabilization or a slight attenuation of the treatment effect. The magnitude of reduction progressively increased during the first 35 weeks, reaching a maximum effect around week 35. In contrast, the weight-loss effect of ecnoglutide exhibited a significant linear time-dependent relationship; as the intervention period lengthened, the reduction in body weight relative to baseline continued to increase steadily, with no apparent plateau or rebound trend observed (**Figures 10**–**12**).

Moderate to substantial heterogeneity was observed for several efficacy outcomes. Leave-one-out sensitivity analyses were performed to assess whether individual studies substantially influenced the pooled estimates. Sequential exclusion of individual studies did not materially alter the overall effect sizes, suggesting that the pooled results were robust. Because only three randomized controlled trials were included, comprehensive subgroup analyses based on baseline characteristics, disease duration, or study design were not feasible. Nevertheless, several clinical and methodological factors may have contributed to the observed heterogeneity. First, differences in comparator interventions (placebo versus dulaglutide) may have influenced the magnitude of treatment effects, as placebo-controlled studies evaluate absolute efficacy whereas active-controlled studies assess comparative effectiveness. Second, variations in ecnoglutide dose regimens (0.4–1.2 mg/week) and follow-up duration may have affected glycemic and weight-related outcomes. Furthermore, differences in baseline HbA1c levels, diabetes duration, and body weight among participants may have contributed to variability in treatment responses.

## 4 Discussion

As a novel glucagon-like peptide-1 receptor agonist (GLP-1 RA), ecnoglutide was the focus of this study, which aimed to evaluate its efficacy and safety in the treatment of type 2 diabetes (19). The study included three randomized controlled trials involving a total of 977 participants. The results showed that, compared with placebo, ecnoglutide significantly reduced HbA1c (%), fasting blood glucose, postprandial blood glucose, 7-point self-monitored blood glucose levels, and body weight. Furthermore, ecnoglutide demonstrated superior glycemic control compared with the active control drug, dulaglutide. Furthermore, the rates at which participants in the ecnoglutide group achieved HbA1c levels <7%, <6.5%, and <5.7% were all higher than those in the placebo and dulaglutide groups. Collectively, these findings support the glycemic-lowering efficacy of ecnoglutide in adults with type 2 diabetes. However, these findings should be interpreted with caution because they are based on only three randomized controlled trials involving a relatively small number of participants.

This study further evaluated the overall safety and tolerability of ecnoglutide. Notably, the overall incidence of adverse events in the ecnoglutide group was higher than that in the placebo group, with gastrointestinal reactions being the most common. This finding is highly consistent with the known safety profile of GLP-1 receptor agonists (20, 21). GLP-1 receptor agonists exert their hypoglycemic and weight-loss effects by activating GLP-1 receptors, which are widely distributed in the central nervous system and peripheral organs such as the pancreas and gastrointestinal tract (22),their effects on gastrointestinal motility are considered one of the core mechanisms of action for this class of drugs. Specifically, GLP-1 receptor agonists slow gastric emptying and inhibit gastric motility; these effects help delay the postprandial absorption of nutrients, thereby improving glycemic control, but they also increase the risk of gastrointestinal adverse events such as nausea, vomiting, diarrhea, and constipation (23, 24). Previous studies have reported that the incidence of gastrointestinal symptoms in patients treated with GLP-1 receptor agonists can reach over 40%, with nausea occurring in approximately 11%–23% of patients, vomiting in approximately 3%–12%, and diarrhea in approximately 5%–12%. There are some differences in the incidence of gastrointestinal adverse reactions among different GLP-1 receptor agonists; however, overall, these reactions are often dose-dependent and are particularly pronounced during the initial phase of treatment and when the dose is increased. The vast majority of patients are able to tolerate them (20, 23, 25). The above results suggest that ecnoglutide, as a novel metabolic therapy with potential for lowering blood glucose and promoting weight loss, exhibits pharmacological characteristics similar to those of other GLP-1 receptor agonists, such as semaglutide and dulaglutide. However, due to the limited number of included studies, the analysis results exhibit heterogeneity; therefore, when interpreting the conclusions, attention must be paid to the impact of confounding factors such as the study population, control groups, dosing regimens, and follow-up duration.

The results of the dose-response analysis show that efficacy indicators such as HbA1c (%), fasting blood glucose, and body weight all exhibit a trend toward improvement as the dose increases. Pharmacological effects depend on the binding of the drug to its receptor; when the receptor has not yet reached saturation, the effect increases with rising dose, which has a sound pharmacological basis (26, 27). GLP-1 receptor agonists bind to GLP-1 receptors on the surface of target tissues, such as pancreatic β-cells, to activate downstream signaling pathways that promote glucose-dependent insulin secretion; their clinical effects are closely related to receptor occupancy (28, 29). Takayanagi et al. (26) found that GLP-1 receptor agonists at commonly used doses require occupation of only 1.1% to 10.7% of GLP-1 receptors to produce significant clinical effects, suggesting that the effect increases with dose within this range of receptor occupancy. In addition, Chen et al. (30) found through a network meta-analysis that several GLP-1 receptor agonists exhibited significant nonlinear dose-response relationships in terms of reducing HbA1c and body weight, confirming that higher doses yield greater clinical benefits as a class effect. An analysis by Yao et al. (31) also demonstrated that GLP-1 receptor agonists are highly effective in controlling blood glucose and managing weight, but it also noted that caution is warranted regarding the increased risk of gastrointestinal adverse events at high doses. It is worth noting that, a dose-response study conducted by Torekov et al. (32) in patients with T2DM using continuous subcutaneous infusion of exogenous GLP-1 directly confirmed that the blood glucose-lowering effect of GLP-1 exhibits a continuous, dose-dependent increase across a wide dose range; the reduction in fasting blood glucose levels gradually increased from 22.7 mg/dL in the low-dose group to 76.2 mg/dL in the high-dose group.

Analysis of time-dependent effects revealed that ecnoglutide exhibited distinct nonlinear time-dependent effects on HbA1c, FPG, PPG, and 7-point self-monitored blood glucose (SMBG), with the magnitude of reduction peaking at approximately 35 weeks, whereas its effect on body weight exhibited a linear time-dependent pattern. This differential pattern is highly consistent with the pharmacokinetic profiles of existing GLP-1 receptor agonists. The “plateau effect” in glycemic control may stem from the self-limiting enhancement of GLP-1 receptor-mediated insulin secretion as blood glucose levels approach normal ranges, as well as the gradual saturation of compensatory improvements in β-cell function. The sustained weight loss is associated with the persistent effects of GLP-1 receptor agonists on the hypothalamic appetite regulation circuit and the long-term cumulative effects of a negative energy balance. Previous studies have also shown that the blood glucose-lowering effect of semaglutide plateaus at approximately 30 weeks, while the weight-loss effect can persist for more than 52 weeks (33). Although one study found that long-term treatment with GLP-1 receptor agonists may reach a weight-loss plateau due to the dynamic narrowing of the energy intake-expenditure gap (34), however, no clear plateau has been observed for ecnoglutide within the current follow-up period, suggesting that this drug may have more sustained weight-loss potential. The temporal decoupling of the blood glucose-lowering and weight-loss curves suggests that ecnoglutide can continue to provide weight-loss benefits even after blood glucose control has stabilized, offering significant therapeutic value for overweight or obese patients with diabetes.

The clinical relevance of ecnoglutide should be interpreted within the rapidly evolving landscape of incretin-based therapies, particularly considering the emergence of highly effective agents such as semaglutide and tirzepatide. Semaglutide, a long-acting GLP-1 receptor agonist administered once weekly, has demonstrated substantial reductions in HbA1c and body weight across the SUSTAIN clinical program (35). Moreover, the SUSTAIN-6 cardiovascular outcomes trial demonstrated that semaglutide reduced the risk of major adverse cardiovascular events in patients with T2DM at high cardiovascular risk, further establishing its position in contemporary diabetes management (36). Compared with semaglutide, ecnoglutide demonstrated a numerically comparable magnitude of glycemic improvement and weight reduction in the present meta-analysis; however, such comparisons should be interpreted cautiously because direct head-to-head randomized trials between these agents are currently unavailable. Differences in baseline HbA1c, patient characteristics, background therapies, and follow-up duration may substantially influence indirect comparisons.

Tirzepatide represents another major advance in incretin-based therapy as a dual glucose-dependent insulinotropic polypeptide (GIP)/GLP-1 receptor agonist. In the SURPASS program, tirzepatide achieved greater reductions in HbA1c and body weight compared with several established glucose-lowering therapies, including semaglutide in the SURPASS-2 trial (37). Furthermore, the SURMOUNT-1 trial demonstrated marked weight reduction with tirzepatide in individuals with obesity, highlighting its expanding role in metabolic disease management (38). The enhanced metabolic effects of tirzepatide may result from complementary activation of GIP and GLP-1 signaling pathways, which may provide additional benefits through coordinated regulation of insulin secretion, appetite control, and energy metabolism. Nevertheless, the clinical positioning of ecnoglutide should not be determined solely by the magnitude of weight loss or HbA1c reduction. As a selective GLP-1 receptor agonist, ecnoglutide has demonstrated dose-dependent efficacy, predictable glucose-lowering effects, and a safety profile consistent with the therapeutic class. Future head-to-head trials comparing ecnoglutide with semaglutide, tirzepatide, and other next-generation incretin therapies are required to clarify their relative efficacy, tolerability, cardiovascular effects, and long-term clinical value.

## 5 Conclusion

In conclusion, this systematic review and meta-analysis suggests that ecnoglutide provides significant improvements in glycemic control and body weight reduction in adults with type 2 diabetes compared with placebo or active control treatments. Ecnoglutide demonstrated dose-dependent improvements in HbA1c, fasting plasma glucose, and body weight, while its glucose-lowering effects appeared to reach a plateau over time. Although ecnoglutide was associated with a higher incidence of adverse events, mainly gastrointestinal events, no significant increase in serious adverse events was observed. However, the current evidence remains limited by the small number of available randomized controlled trials and relatively short follow-up durations. Therefore, larger multicenter randomized trials with longer observation periods are warranted to further establish the long-term efficacy, safety, and clinical applicability of ecnoglutide in diverse populations with type 2 diabetes.

## 6 Limitations and future directions

This meta-analysis has several limitations that cannot be overlooked. First, the analysis included only three randomized controlled trials, involving a total of 977 participants. The insufficient sample size significantly reduced statistical power, affected the precision of the pooled effect size, and made it impossible to conduct a reliable assessment of rare adverse events (39). Furthermore, because the number of included trials was relatively small, the pooled results may be susceptible to evidence fragility and small-study effects, potentially leading to an overestimation of treatment efficacy. In addition, the limited number of studies prevented comprehensive subgroup analyses to further explore potential sources of heterogeneity, and formal assessment of publication bias using funnel plots or Egger’s test was not performed because fewer than 10 studies were available. Although selective publication cannot be completely excluded, this limitation should be considered when interpreting the findings.

Second, one of the included studies was an open-label design, which introduced unavoidable performance and detection biases; the lack of blinding may have influenced subjective outcomes, such as weight-related behaviors, dietary adherence, and patient-reported adverse events particularly gastrointestinal symptoms thereby potentially exaggerating treatment effects or safety signals.

Third, the study populations were relatively homogeneous; most trials recruited patients with a short duration of diabetes and excluded those with advanced renal impairment or significant cardiovascular comorbidities, which limited our ability to determine the external validity and generalizability of the findings to a broader, higher-risk real-world population.

Finally, most included studies had relatively short follow-up periods, limiting the assessment of long-term cardiovascular safety, durability of glycemic control, sustained weight reduction, and rare safety outcomes. Future long-term outcome trials are therefore required to clarify the durability of efficacy and the long-term risk profile of ecnoglutide.

Based on these limitations, large-scale, multicenter randomized controlled trials are warranted to increase statistical power and provide more robust estimates of the efficacy and safety of ecnoglutide. Future studies should include more diverse patient populations, including individuals with longer diabetes duration, higher cardiovascular risk, and different comorbidity profiles, to improve external validity and assess treatment effects across clinically relevant subgroups.

Moreover, adequately powered comparative trials against established GLP-1 receptor agonists, such as semaglutide, and dual incretin receptor agonists, such as tirzepatide, are needed to better define the relative clinical benefits of ecnoglutide. Longer outcome-oriented trials should also focus on clinically meaningful endpoints, including durability of glycemic control, long-term weight trajectories, major adverse cardiovascular events, and rare safety outcomes.

In addition, real-world evidence from large observational cohorts combined with pharmacoeconomic evaluations will be valuable for assessing treatment adherence, long-term effectiveness, healthcare utilization, and cost-effectiveness of ecnoglutide in routine clinical practice. These efforts will provide more comprehensive evidence to guide personalized treatment strategies for patients with type 2 diabetes.

## Data Availability

The original contributions presented in the study are included in the article/**Supplementary Material**. Further inquiries can be directed to the corresponding authors.
