# Supplementary material for: Dose-response and time-course effects of ecnoglutide in type 2 diabetes: a systematic review and meta-analysis of efficacy
Source: Front Endocrinol (Lausanne). 2026 Sep 11;17:1934027. doi: 10.3389/fendo.2026.1934027 (PMC13612292; doi:10.3389/fendo.2026.1934027)
Supplement: Supplementary file 1 [file Table1.docx]

**Supplementary Material**

**Supplementary Table 1: Search Strategy**

| PubMed (n=9) | "ecnoglutide"[All Fields] AND ("diabete"[All Fields] OR "diabetes mellitus"[MeSH Terms] OR ("diabetes"[All Fields] AND "mellitus"[All Fields]) OR "diabetes mellitus"[All Fields] OR "diabetes"[All Fields] OR "diabetes insipidus"[MeSH Terms] OR ("diabetes"[All Fields] AND "insipidus"[All Fields]) OR "diabetes insipidus"[All Fields] OR "diabetic"[All Fields] OR "diabetics"[All Fields] OR "diabets"[All Fields]) |
| --- | --- |
| Cochrane Library (n=13) | ecnoglutide |
| Embase  (n=36) | (ecnoglutide) AND (diabetes) |
| Web of Science  (n=19) | ecnoglutide |

**Supplementary Table 2: List of excluded studies and corresponding exclusion reasons during literature screening**

| Studies excluded | Reasons |
| --- | --- |
| Ji, 2025（1） | Participants did not meet the predefined inclusion criteria |
| Richard Friend, 2021（2） | Participants did not meet the predefined inclusion criteria |
| Linong Ji, 2023（3） | Participants did not meet the predefined inclusion criteria |
| Wei Zhao, 2025（4） | Participants did not meet the predefined inclusion criteria |
| Linong Ji, 2025（5） | Participants did not meet the predefined inclusion criteria |
| Junaidi, 2024（6） | Participants did not meet the predefined inclusion criteria |
| - Richard Friend, 2021（7） | Participants did not meet the predefined inclusion criteria |
| **Jintong Li, 2025（8）** | Participants did not meet the predefined inclusion criteria |
| Zhu, 2023（9） | Participants did not meet the predefined inclusion criteria |
| Zhu, 2024（10） | Participants did not meet the predefined inclusion criteria |
| Xiaoying Li, 2022（11） | Overlapping or duplicate study population with already included studies |
| Dalong Zhu, 2022（12） | Overlapping or duplicate study population with already included studies |
| Xu, 2024（13） | Overlapping or duplicate study population with already included studies |
| Zhu, 2024（14） | Overlapping or duplicate study population with already included studies |
| Qin Yu, 2023（15） | Not a randomised controlled trial (RCT) |
| Guo, 2023（16） | Not a randomised controlled trial (RCT) |

**Supplementary Table 3: Risk of Bias Assessment**

| Study | Randomization process | Deviations from intended interventions | Missing outcome data | Measurement of the outcome | Selection of the reported result | Overall Bias |
| --- | --- | --- | --- | --- | --- | --- |
| Dalong Zhu2024 | Low | Low | Low | Low | Low | Low |
| Dalong Zhu2026 | Low | Low | Low | Low | Low | Low |
| Yang He2025 | Low | Some concerns | Low | Low | Low | Some concerns |
